# Abdominal wall schwannoma: a case report 

**Published:** 2020

**Authors:** Mohamed Tarchouli, Mohamed Essarghini, Ouadie Qamouss, Abdennasser El Kharras, Ahmed Bounaim

**Affiliations:** 1 *Department of Surgery, First Medical and Surgical Center, Agadir, Morocco*; 2 *Faculty of Medicine and Pharmacy, Sidi Mohamed Ben Abdellah University, Fez, Morocco*; 3 *Department of Visceral Surgery, Mohammed V Military Teaching Hospital, Rabat, Morocco*; 4 *Faculty of Medicine and Pharmacy, Mohammed V University, Rabat, Morocco*; 5 *Laboratory of Pathology Souss, Agadir, Morocco*; 6 *Department of Radiology, First Medical and Surgical Center, Agadir, Morocco*; 7 *Faculty of Medicine and Pharmacy, Mohammed V University, Rabat, Morocco*

**Keywords:** Peripheral nerve sheath tumors, Schwannoma, Abdominal wall

## Abstract

Schwannomas or neurilemmomas are benign and slow-growing tumors that arise exclusively from Schwann cells in peripheral nerve sheaths. These neoplasms theoretically can occur anywhere in the body, but they most frequently affect extremities, as well as head and neck region. However, their presentation in the abdominal wall is extremely rare and only few cases have been reported in the literature. Subcutaneous lesions may be asymptomatic and only incidentally discovered upon physical examination or imaging. However, occasionally they induce mass effects on surrounding large nerves. We present the case of a 34-year-old man with abdominal wall pain localized in the right iliac fossa and palpable subcutaneous mass. Ultrasound and CT scan revealed a solid well-defined mass of the abdominal wall. Following surgical excision under general anesthesia, histological examination was consistent with the diagnosis of benign schwannoma.

## Introduction

 Schwannoma or neurilemmoma is an uncommon benign tumor arising from the Schwann cells in the peripheral nerve sheath (1, 2). Most frequently, it is discovered incidentally and affects extremities, as well as head and neck region. However, occurrence in the abdominal wall is extremely rarely encountered (3-5). Through this work, we present the eighth case ever reported, to the best of our knowledge, in the English medical literature. 

## Case Report

A 34-year-old man was admitted to our institution for chronic and intermittent abdominal wall pain localized in the right iliac fossa. This pain, evolving for the past one year, was not related to any particular position, oral feeding, or bowel movements. There was no history of weight loss or any gastrointestinal symptoms such as nausea-vomiting, diarrhea-constipation, or signs of gastrointestinal bleeding. He denied any abdominal trauma or past surgical operations.

On physical examination, a well-defined subcutaneous mass was palpable in the right iliac fossa. The mass was firm, non-tender, and not fixed to the skin or the muscular plane of the abdominal wall. It was sensitive to both light touch and deep pressure. No peritoneal signs or overlying skin changes were found. All routine laboratory tests including blood cell count, CRP (C-reactive protein), serum electrolytes, and hemostasis tests were completely unremarkable. Tumor markers including carcinoembryonic antigen and carbohydrate antigen (CA 19-9) were within the normal ranges.

**Figure 1:**
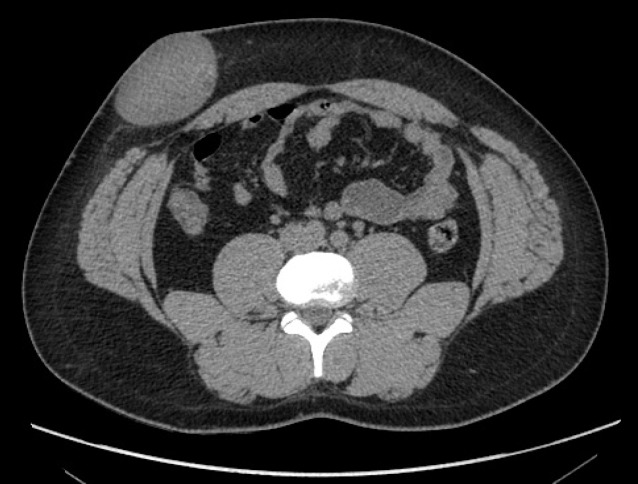
Basal abdominal CT scan revealing an oval-shaped, homogenous mass, with muscular density, developed in the subcutaneous tissues of the anterior abdominal wall. The mass is well-circumscribed with regular limits clearly differentiated from the surrounding structures

**Figure 2:**
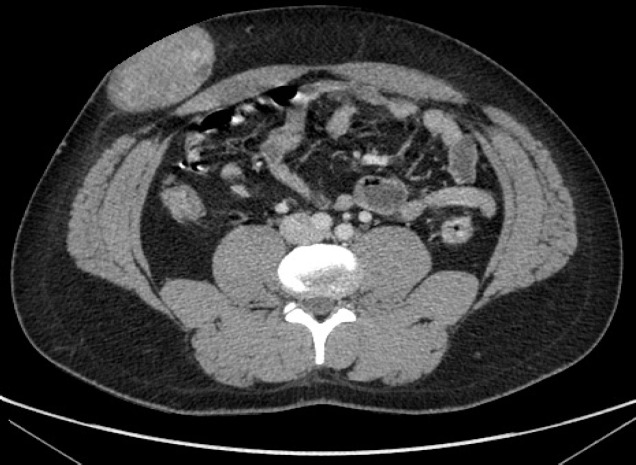
Post-contrast abdominal CT scan (later phase) revealing a mild and homogeneous enhancement with a few scattered liquid chambers

An abdominal ultrasound examination was performed, which showed a solid and heterogeneous mass developed in the right abdominal wall. A basal computed tomography (CT) revealed a 7×5 cm well-circumscribed tissue-mass located in the subcutaneous plane of the right iliac fossa (Figure 1). After intravenous administration of contrast media, the lesion showed a modest and homogeneous enhancement with a few scattered liquid chambers (figure 2). No lesions in the peritoneal cavity or parietal defects were identified. 

**Figure 3:**
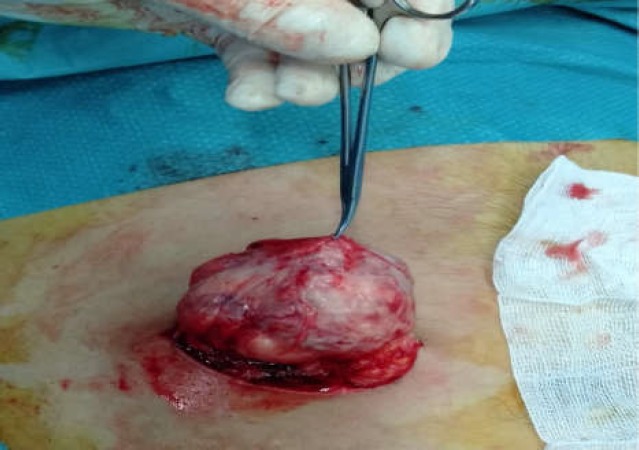
Operative view showing a well-defined subcutaneous mass easily enucleated through a selective incision in the right iliac fossa

Surgical resection of the mass was proposed and the patient was then operated under general anesthesia. The mass was easily enucleated after a direct anterior approach by a selective incision in the right iliac fossa (figure 3). 

**Figure 4:**
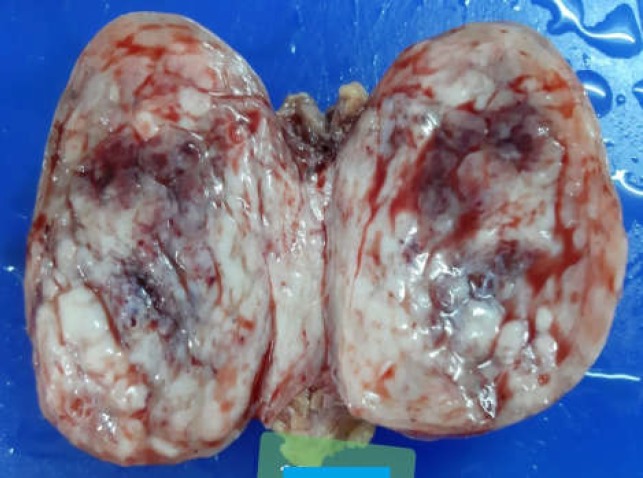
Macroscopic view of the resected specimen showing an encapsulated solid tumor with heterogeneous appearance and the presence of hemorrhagic as well as mucoid changes

It was exclusively involving the subcutaneous plane without any connection to the musculofascial layer or the parietal peritoneum of the abdominal wall. Pathological examination of the resected specimen revealed an encapsulated mass measuring 7x7x5 cm with heterogeneous appearance and presence of multiple areas of hemorrhagic and mucoid changes. Macroscopically, no obvious necrosis was observed (figure 4). 

**Figure 5:**
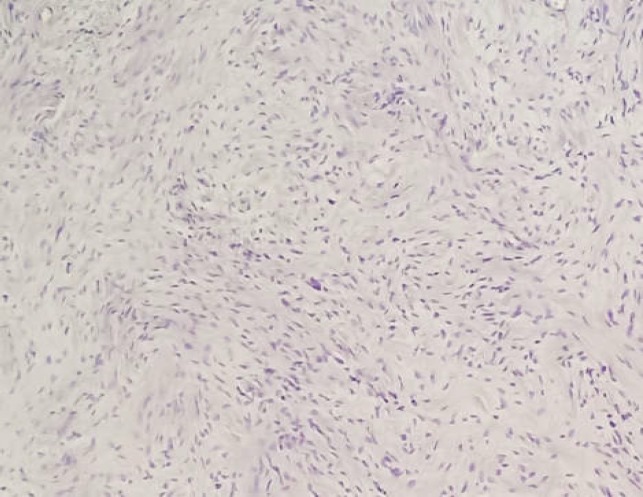
Microscopic findings: Tumor proliferation composed of spindle-shaped cells with a benign appearance. No evidence of mitosis or cytonuclear atypia (hematoxylin-eosin staining, original magnification ×10)

**Figure 6:**
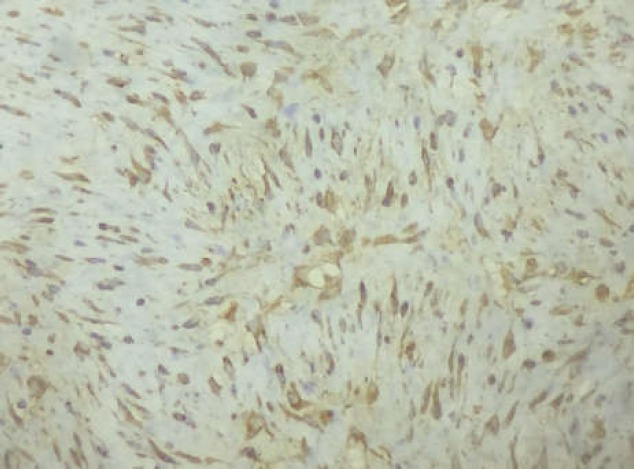
Immunohistochemical study showing strong and diffuse staining for the S-100 protein. (immunohistochemistry, original magnification ×40)

Microscopically, the tumor was composed of proliferating spindle cells arranged in bundles without cytonuclear atypia or abnormal mitoses (Figure 5). Immunohistochemistry showed that the tumor-cells were strongly and diffusely positive for S100 protein, which is consistent with the diagnosis of benign schwannoma (Figure 6). The patient was discharged one day after surgery without any postoperative complications. 

## Discussion

Peripheral nerve sheath tumors are a heterogeneous group of neoplasms that originate from different components of the peripheral nerve sheath (Schwann cells, perineurial cells, and fibroblasts). These neoplasms can be divided into two separate groups with different histologic features: benign tumors mainly represented by schwannomas and neurofibromas and malignant tumors grouped under the term of malignant peripheral nerve sheath tumors. Benign and malignant peripheral nerve sheath tumors may develop sporadically or as part of neurofibromatosis (2, 4, 6). 

Schwannomas are benign, encapsulated, slow-growing tumors that arise exclusively from Schwann cells in neural sheaths (7-9). Theoretically, schwannomas can occur anywhere in the body, but they most frequently affect extremities, as well as head and neck region. They can also infrequently occur in other locations including retroperitoneum, pelvis, perineum, mediastinum, and gastrointestinal tract (1, 10). Nevertheless, their presentation in the abdominal wall is extremely rare and only few cases have been reported in the medical literature. We searched English-language manuscripts in PubMed/Medline database using the following key-words: schwannoma, abdominal wall. We found only 7 other case reports (Table 1). 

These neoplasms can present at any age, and most commonly occur between 40 and 60 years of age (8). There is no known predilection based on gender or race. The vast majority of schwannomas are seen sporadically as single isolated lesions, but they can also occur, usually as multiple lesions, as part of neurofibromatosis diseases. Indeed, bilateral vestibular schwannomas are typical for neurofibromatosis type 2. More recently schwannomatosis is recognized as the third major form of neurofibromatosis which causes multiple schwannomas without vestibular tumors (2, 11-14). Schwannomas do not occur in neurofibromatosis type 1.

In peripheral topography, most schwannomas arise from the nerve sheath of large peripheral nerves and occur at the level of the subcutaneous plane (1, 10). Typically, they do not affect the nerve conduction and present as an asymptomatic slow growing mass. However, because of their progressive development, they can give signs of nerve compression such as pain and dysesthesia (4, 5). Thus, the clinical signs are variable and depend on the tumor location. Subcutaneous lesions may be asymptomatic and only incidentally discovered on physical examination or imaging. 

**Table 1:** Abdominal wall schwannoma reported in the English-language literature

Authors Year Country	Age Gender	Location	symptoms	Imaging Findings	Pathological features
Khorgami et al. (2009) (Iran)^[14]^	28/F	Right side of abdomen	Abdominal distention and sustained pain for 2 months with a medical history of NF1	US and CT: heterogenic solid mass in RUQ of abdomen that extended down to the RLQ of the abdomen	Atypical spindle cells with mild pleomorphism and high mitotic activity that suggest malignant peripheral nerve sheet tumor. Tumor size: 16×13×6 cm Final diagnosis: malignant schwannoma
Bhatia et al. (2010) (UK)^[1]^	64/F	Right iliac fossa	Asymptomatic. Incidentally identified on a private whole-body “screening” CT	CT with contrast: heterogeneous mass in right iliac fossa adjacent to the peritoneal wall	Solid tumor composed of chronic inflammatory cells and spindle cells arranged on hypocellular and hypercellular areas. The spindle cells showed hyperchromatic and irregular nuclei although no mitotic figures were seen. Immunohistochemistry showed a strong staining for S100 protein. Tumor size: 6×4×3.5 cm Final diagnosis: benign ancient schwannoma
Mishra et al. (2013) (Libya)^[4]^	29/F	Anterior left upper abdominal wall	Painless lump gradually increasing in size over 10 months	US: heterogeneous well-encapsulated mass, hypovascular on color flow. MRI: solid mass, hypointense on T1, heterogeneously hyperintense on T2 with fat suppressed sequences suggesting cystic degeneration	Solid tumor composed of spindle cells arranged on hypocellular and hypercellular areas. The spindle cells showed nuclear palisading although no mitotic figures were seen. Immunohistochemistry showed a strong staining for S100 protein. Tumor size: 6 cm in diameter Final diagnosis: benign ancient schwannoma
Balzarotti et al. (2014) (Switzerland)^[7]^	57/F	Left lower quadrant of the abdominal wall	Well-localized parietal pain lasting for 3 years, without palpable mass	US: Well-defined cystic painless lesion located in the muscular layer of the LLQ. CT: Well-demarcated homogeneous mass with a modest and homogeneous enhancement after administration of contrast media.	Solid tumor composed of spindle cells arranged on hypocellular and hypercellular areas. No significant atypia were seen. Immunohistochemistry showed a strong staining for S100 protein. Tumor size: 2 cm in diameter Final diagnosis: benign schwannoma
Liu et al. (2014) (China)^[3]^	67/F	Right anterior abdominal wall	Painless mass for 10 year, gradually increasing in size and becoming painful over 1 year	US: welldefined heterogeneous mass. CT: solid homogeneous, low-density mass with gradual and heterogeneous enhancement in the arterial and venous phases.	Solid tumor composed of abundant spindle-shaped cells, which locally invaded the surrounding fat tissues. Immunohistochemistry revealed a negative staining for S100 protein. Tumor size: 5.6 cm in diameter. Final diagnosis: schwannoma of low malignant potential.
Ginesu et al. (2016) (Italy)^[13]^	62/F	Right iliac fossa	Abdominal pain with palpable mass	US: hypoechoic mass. CT: well-circumscribed mass with internal calcifications and little contrast enhancement in late phase.	Histological features of schwannoma. Tumor size: 8x3.3 x4.2-cm. Final diagnosis: ancient schwannoma.
Lam et al. (2019) (USA)^[2]^	70/M	Left lower quadrant of the abdominal wall	Abdominal pain with palpable mass	US: solid heterogeneously hypoechoic mass with mild internal vascularity	Solid tumor composed of spindle cells arranged on hypocellular and hypercellular areas. Occasionally mitotic figures were seen. Immunohistochemistry showed a strong staining for S100 protein. Tumor size: 0.9x0.9x1cm. Final diagnosis: benign schwannoma

However, they occasionally produce pressure effects on surrounding large nerves, as found in our patient. 

Although schwannomas may be imaged by ultra-sound or computed tomography, magnetic resonance imaging (MRI) is the most reliable means of defining these tumors. On CT scan, schwannomas usually appear as a well-defined homogeneous softtissue mass wich is iso to hypodense compared to muscular structures.. The lesions show minimal or mild heterogeneous enhancement after the administration of contrast agent. On MRI, schwannomas appear as hypointense on T1-weighted and hyperintense on T2-weighted images. Typically, small schwannomas tend to enhance uniformly after gadolinium while larger lesions show a more heterogeneous enhancement (4, 15, 16). Longstanding schwannomas once called “Ancient” schwannomas can show degenerative changes, such as calcifications, hyanilinization, and cystic cavitation; findings that can be identified on imaging studies (5, 16). In the present case, no cyst formation, calcification, or necrosis was found.

In pathology analysis, most schwannomas are unilobular masses surrounded by fibrous capsule derived from epineurium of the involved nerve. The identification of a nerve entering and exiting a mass is suggestive for a peripheral nerve sheath tumor, while the eccentric association with the nerve trunk is considered to be pathognomonic for schwannoma (17). Microscopically, these lesions are composed of spindle cells with nuclear palisading patterns (Verocay bodies) and biphasic architecture of dense (Antoni A) and loose (Antoni B) areas (2, 18, 19). The term “ancient” is used to describe long-standing schwannoma with degenerative changes including cyst formation, calcification, interstitial fibrosis, hemosiderin deposition, and vascular hyaline degeneration (5, 10, 20). A suspicion for malignancy may be discussed on the presence of hyperchromatic cells and nuclear atypia. Diffuse and strong immunoreactivity for S100 protein is characteristic of schwannomas (19, 21, 22). Immunohistochemistry can be used to aid diagnosis and to differentiate them from other nerve sheath tumors.

The optimal treatment for benign schwannoma is complete surgical excision with intact margins. The prognosis of these lesions is good, the recurrence is unusual, and the malignant degeneration is exceedingly rare (23, 24). Schwannoma of the abdominal wall should be considered as possible diagnosis in patients with abdominal mass.

## References

[1] Bhatia RK, Banerjea A, Ram M, Lovett BE (2010). Benign ancient schwannoma of the abdominal wall: an unwanted birthday present. BMC Surg.

[2] Lam R, Hunt BL, Arreola-Owen O (2019). Abdominal Wall Schwannoma. Fed Pract.

[3] Dane B, Dane C, Basaran S, Erginbas M, Cetin A (2010). Vaginal Schwannoma in a case with uterine myoma. Ann Diagn Pathol.

[4] Liu Y, Chen X, Wang T, Wang Z (2014). Imaging observations of a schwannoma of low malignant potential in the anterior abdominal wall: A case report. Oncol Lett.

[5] Mishra A, Hamadto M, Azzabi M, Elfagieh M (2013). Abdominal wall schwannoma: case report and review of the literature. Case Rep Radiol.

[6] Bouvier C, Maues de Paula A, Roche PH, Chagnaud C, Figarella-Branger D (2013). Tumors of the peripheral nervous system. EMC Neurol.

[7] Kim DH, Murovic JA, Tiel RL, Moes G, Kline DG (2005). A series of 397 peripheral neural sheath tumors: 30-year experience at Louisiana State University Health Sciences Center. J Neurosurg.

[8] Le Guellec S (2015). Nerve sheath tumours. Ann Pathol.

[9] Shelat VG, Li K, Naik S, Ng CY, Rao N, Rao J (2013). Abdominal schwannomas: case report with literature review. Int Surg.

[10] Balzarotti R, Rondelli F, Barizzi J, Cartolari R (2015). Symptomatic schwannoma of the abdominal wall: A case report and review of the literature. Oncol Lett.

[11] Asthagiri AR, Parry DM, Butman JA, Kim HJ, Tsilou ET, Zhuang Z (2009). Neurofibromatosis type 2. Lancet.

[12] Evans DG, Bowers NL, Tobi S, Hartley C, Wallace AJ, King AT (2018). Schwannomatosis: a genetic and epidemiological study. J Neurol Neurosurg Psychiatry.

[13] MacCollin M, Chiocca EA, Evans DG, Friedman JM, Horvitz R, Jaramillo D (2005). Diagnostic criteria for schwannomatosis. Neurology.

[14] Merker VL, Esparza S, Smith MJ, Stemmer-Rachamimov A, Plotkin SR (2012). Clinical features of schwannomatosis: a retrospective analysis of 87 patients. Oncologist.

[15] Crist J, Hodge JR, Frick M, Leung FP, Hsu E, Gi MT (2017). Magnetic Resonance Imaging Appearance of Schwannomas from Head to Toe: A Pictorial Review. J Clin Imaging Sci.

[16] Pilavaki M, Chourmouzi D, Kiziridou A, Skordalaki A, Zarampoukas T, Drevelengas A (2004). Imaging of peripheral nerve sheath tumors with pathologic correlation: pictorial review. Eur J Radiol.

[17] Beaman FD, Kransdorf MJ, Menke DM (2004). Schwannoma: radiologic-pathologic correlation. Radiographics.

[18] Guo A, Liu A, Wei L, Song X (2012). Malignant peripheral nerve sheath tumors: differentiation patterns and immunohistochemical features - a mini-review and our new findings. J Cancer.

[19] Rodriguez FJ, Folpe AL, Giannini C, Perry A (2012). Pathology of peripheral nerve sheath tumors: diagnostic overview and update on selected diagnostic problems. Acta Neuropathol.

[20] Klijanienko J, Caillaud JM, Lagacé R (2006). Cytohistologic correlations in schwannomas (neurilemmomas), including “ancient,” cellular, and epithelioid variants. Diagn Cytopathol.

[21] Shu Z, Li C, Sun M, Li Z (2019). Intestinal Schwannoma: A clinicopathological, immunohistochemical, and prognostic study of 9 cases. Gastroenterol Res Pract.

[22] Wei S, Henderson-Jackson E, Qian X, Bui MM (2017). Soft Tissue Tumor Immunohistochemistry Update: Illustrative Examples of Diagnostic Pearls to Avoid Pitfalls. Arch Pathol Lab Med.

[23] Ginesu GC, Puledda M, Feo CF, Cossu ML, Fancellu A, Addis F (2016). Abdominal Wall Schwannoma. J Gastrointest Surg.

[24] Khorgami Z, Nasiri S, Rezakhanlu F, Sodagari N (2009). Malignant schwannoma of anterior abdominal wall: report of a case. J Clin Med Res.

